# Supplementary material for: Real life condition evaluation of Inoserp PAN-AFRICA antivenom effectiveness in Cameroon
Source: PLoS Negl Trop Dis. 2023 Nov 8;17(11):e0011707. doi: 10.1371/journal.pntd.0011707 (PMC10659212; doi:10.1371/journal.pntd.0011707)
Supplement: S1 Appendix — (DOCX) [file pntd.0011707.s001.docx]

**Appendix 1: Management algorithm recommended by Cameroonian Ministry of envenomation patients**

**
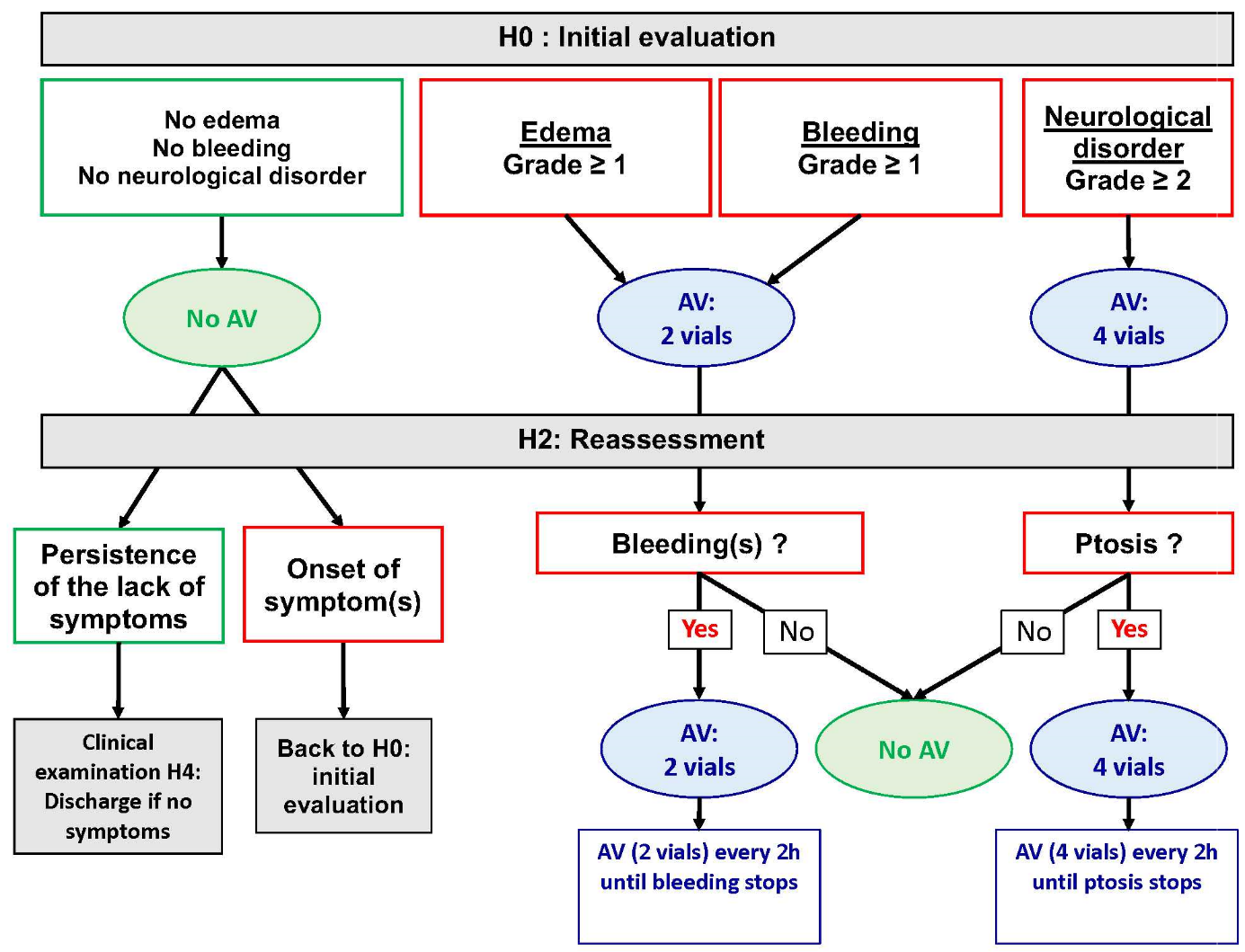
**

**Gradation of edema:**

**0. No edema**

**1. Localized edema not exceeding the nearest joint**

**2. Progressive edema not exceeding 2 contiguous joints**

**3. Extensive edema not exceeding the root of the limb**

**4. Edema extending beyond the root of the limb (hydrops)**

**Gradation of bleeding:**

**0. No bleeding**

**1. Persistent local bleeding at fang marks for more than one hour**

**2. Bleeding from the gums, nose, scars and recent wounds**

**3. Ecchymosis, hematoma, purpura, phlyctens**

**4. Internal hemorrhage (peritoneal, meningeal, metrorrhagia, hematemesis, etc.)**

**Gradation of neurological disorders:**

**0. No neurological disorder**

**1. Local anesthesia, tingling affecting the bitten limb**

**2. Profuse sweat, saliva and vomiting, miosis**

**3. Bilateral ptosis (± speech, vision, hearing and/or swallowing disorders)**

**4. Respiratory distress, impossibility to communicate**
