# Supplementary material for: Real life condition evaluation of Inoserp PAN-AFRICA antivenom effectiveness in Cameroon
Source: PLoS Negl Trop Dis. 2023 Nov 8;17(11):e0011707. doi: 10.1371/journal.pntd.0011707 (PMC10659212; doi:10.1371/journal.pntd.0011707)
Supplement: S2 Appendix — (DOCX) [file pntd.0011707.s002.docx]

**Appendix 2: gradation of whole-blood clotting time on dry tube (WBCT)**

1. Sample 2 mL blood in a clean dry glass tube
2. Let the tube stand without shaking it
3. Read the result after 20 and 60 minutes of waiting

**
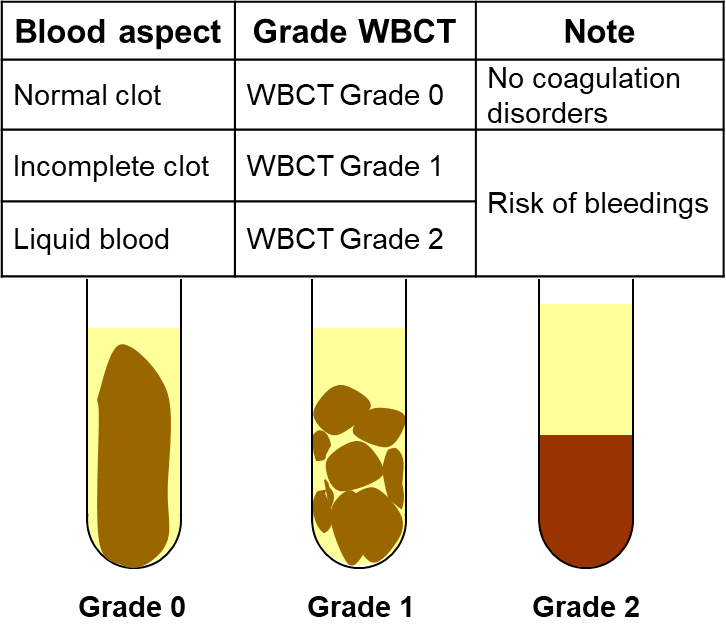
**
