# Supplementary material for: Real life condition evaluation of Inoserp PAN-AFRICA antivenom effectiveness in Cameroon
Source: PLoS Negl Trop Dis. 2023 Nov 8;17(11):e0011707. doi: 10.1371/journal.pntd.0011707 (PMC10659212; doi:10.1371/journal.pntd.0011707)
Supplement: S3 Appendix — (DOCX) [file pntd.0011707.s003.docx]

**Appendix 3: List of snakes for which the IPA is effective** (in bold, snake species present in Cameroon)

Viperidae:

- **Echis ocellatus;**
- Echis leucogaster;
- Echis pyramidum;
- **Bitis arietans;**
- Bitis rhinoceros;
- **Bitis nasicornis;**
- **Bitis gabonica.**

Elapidae:

- **Dendroaspis polylepis;**
- Dendroaspis viridis;
- Dendroaspis angusticeps;
- **Dendroaspis jamesoni;**
- **Naja nigricollis;**
- **Naja melanoleuca;**
- **Naja haje;**
- Naja pallida;
- Naja nubiae;
- **Naja katiensis;**
- Naja senegalensis.
