# Supplementary material for: Real life condition evaluation of Inoserp PAN-AFRICA antivenom effectiveness in Cameroon
Source: PLoS Negl Trop Dis. 2023 Nov 8;17(11):e0011707. doi: 10.1371/journal.pntd.0011707 (PMC10659212; doi:10.1371/journal.pntd.0011707)
Supplement: S4 Appendix — (DOCX) [file pntd.0011707.s004.docx]

**Appendix 4:** **Baseline factors associated with early improvement of bleedings (logistic regression, N=117)**

|  | N | Early improvement (%) | Crude OR (95% CI) | p | Adj. OR (95% CI) | p |
| --- | --- | --- | --- | --- | --- | --- |
| Gender  Male  Female | 66  51 | 40 (60,6)  27 (52,9) | 1  0.73 (0.35-1.53) | 0.41 |  |  |
| Age (in years)  5-11  12-19  20-40  > 40 | 15  22  47  33 | 9 (60,0)  13 (59,1)  28 (59,6)  17 (51,5) | 1.02 (0.31-3.33)  0.98 (0.35-2.75)  1  0.72 (0.29-1.77) | 0.89 |  |  |
| Time since snakebite  [0-2h[  [2h-12h[  [12h-24h[  [24h-48h[  ≥ 48H | 9  44  18  18  28 | 4 (44,4)  27 (61,4)  15 (83,3)  8 (44,4)  13 (46,4) | 0.50 (0.12-2.14)  1  3.15 (0.79-12.51)  0.50 (0.17-1.53)  0.55 (0.21-1.42) | 0.06 |  |  |
| Traditional medicine  No  Yes | 92  25 | 53 (57,6)  14 (56,0) | 1.07 (0.44-2.60)  1 | 0.89 |  |  |
| Treatment before arriving at the center  No  Yes | 31  86 | 18 (58,1)  49 (57,0) | 1.05 (0.46-2.40)  1 | 0.92 |  |  |
| Region  North Cameroon  South-Cameroon | 105  12 | 62 (59.0)  5 (41,7) | 1  0.49 (0.15-1.66) | 0.25 |  |  |
| Glasgow score at admission  <15  15 | 4  113 | 2 (50,0)  65 (57,5) | 0.74 (0.10-5.43)  1 | 0.77 |  |  |
| Edema grading at admission  0-1  2  ≥3 | 29  45  43 | 17 (58,6)  25 (55,6)  25 (58,1) | 1.02 (0.39-2.65)  0.9 (0.39-2.09)  1 | 0.96 |  |  |
| Bleeding grading at admission  1  2  ≥3 | 7  85  25 | 5 (71.4)  45 (52.9)  17 (68.0) | 2.22 (0.40-12.09)  1  1.89 (0.74-4.85) | 0.29 |  |  |
| Neurotoxicity grading at admission  0-1  ≥2 | 111  6 | 65 (58,6)  2 (33,3) | 1  0.35 (0.06-2.01) | 0.23 |  |  |
