# Supplementary material for: Real life condition evaluation of Inoserp PAN-AFRICA antivenom effectiveness in Cameroon
Source: PLoS Negl Trop Dis. 2023 Nov 8;17(11):e0011707. doi: 10.1371/journal.pntd.0011707 (PMC10659212; doi:10.1371/journal.pntd.0011707)
Supplement: S5 Appendix — (DOCX) [file pntd.0011707.s005.docx]

**Appendix 5:** **Factors associated with disappearance of bleedings (AFT model, N = 117)**

| Variables | Crude TR (CI 95%) | p |
| --- | --- | --- |
| Gender  Male  Female | 1  0.92 (0.64-1.31) | 0.64 |
| Age (in years)  [5-11]  [12-19]  [20-40]  > 40 | 1.19 (0.68-2.09)  1.05 (0.64-1.71)  1  1.29 (0.84-1.98) | 0.69 |
| Time since snakebite  [0-2h[  [2h-12h[  [12h-24h[  [24h-48h[  ≥ 48H | **2.01 (0.96-4.17)**  **1.84 (1.11-3.04)**  **1**  **2.79 (1.54-5.08)**  **2.65 (1.54-4.56)** | **0.005** |
| Traditional medicine  Yes  No | 1  0.82 (0.53-1.26) | 0.37 |
| Treatments before arriving at the center  Yes  No | 1  1.12 (0.75-1.70) | 0.59 |
| Region  North Cameroon  South-Cameroon | 1  1.64 (0.92-2.93) | 0.09 |
| Glasgow score at admission  <15  15 | 0.97 (0.36-2.64)  1 | 0.95 |
| Edema grading at admission  0-1  2  ≥3 | 1  1.17 (0.75-1.84)  1.34 (0.85-2.11) | 0.46 |
| Bleeding grading at admission  1  2  ≥3 | 1  0.79 (0.37-1.68)  0.85 (0.55-1.32) | 0.68 |
| Neurotoxicity grading at admission  0-1  ≥2 | 1  1.87 (0.81-4.31) | 0.14 |

TR: Time ratio; CI: confidence interval
