# Supplementary material for: Real life condition evaluation of Inoserp PAN-AFRICA antivenom effectiveness in Cameroon
Source: PLoS Negl Trop Dis. 2023 Nov 8;17(11):e0011707. doi: 10.1371/journal.pntd.0011707 (PMC10659212; doi:10.1371/journal.pntd.0011707)
Supplement: S6 Appendix — (DOCX) [file pntd.0011707.s006.docx]

**Appendix 6: Baseline factors associated with early improvement of neurotoxicity (logistic regression, N = 23)**

| Variables | N | Success, N (%) | Crude OR (95% CI) | p |
| --- | --- | --- | --- | --- |
| Gender  Male  Female | 13  10 | 2 (15.4)  7 (70.0) | 1  12.83 [1.70-97.19] | 0.007 |
| Age (in years)  5-11  12-19  20-40  >40 | 5  3  8  7 | 2 (40.0)  -  3 (37.5)  4 (57.1) | 1.12 [0.14-9.21]  0.22 [0.01-5.80]  1  2.02 [0.29-14.00] | 0.61 |
| BMI (> 20 years) (N= 13)  18.5-24.9  25-39,9  ≥ 40 | 5  2  6 | 3 (60.0)  -  3 (50.0) | 1.4 [0.15-12.60]  0.20 [0.01-5.87]  1 | 0.54 |
| Time since snakebite  [0-2h[  [2h-12h[  [12h-24h[  [24h-48h[  ≥ 48H | 4  11  2  2  4 | 1 (25.0)  6 (54.6)  1 (50.0)  1 (50.0)  - | 0.36 [0.04-3.35]  1  0.84 [0.07-10.64]  0.84 [0.07-10.64]  0.09 [0.00-2.16] | 0.61 |
| Number of initial vials  2  4 | 12  11 | 5 (41.7)  4 (36.4) | 1  0.80 [0.15-4.30] | 0.79 |
| Traditional medicine  Yes  No | 15  8 | 6 (40.0)  3 (37.5) | 1  0.90 [0.15-5.26] | 0.91 |
| Treatment before arriving at the center  Yes  No | 8  15 | 4 (50.0)  5 (33.3) | 2.00 [0.35-11.54]  1 | 0.44 |
| Region  North Cameroon  South Cameroon | 11  12 | 4 (36.4)  5 (41.7) | 1  1.25 [0.23-6.71] | 0.79 |
| Glasgow score at admission  < 15  15 | 9  14 | 3 (33.3)  6 (42.9) | 0.67 [0.12-3.81]  1 | 0.65 |
| Edema before injection  0-1  2  ≥3 | 12  6  5 | 4 (14.3)  4 (60.0)  1 (66.7) | 1  4.00 [0.50-31.98]  0.50 [0.04-6.08] | 0.24 |
| Hemotoxicity before injection  0  1  2 | 17  1  5 | 8 (47.1)  -  1 (20.0) | 1  0.37 [0.01-10.43]  0.37 [0.05-2.94] | 0.58 |

OR: odds ratio; CI: confidence interval; BMI: body mass index
