# Supplementary material for: Real life condition evaluation of Inoserp PAN-AFRICA antivenom effectiveness in Cameroon
Source: PLoS Negl Trop Dis. 2023 Nov 8;17(11):e0011707. doi: 10.1371/journal.pntd.0011707 (PMC10659212; doi:10.1371/journal.pntd.0011707)
Supplement: S7 Appendix — (DOCX) [file pntd.0011707.s007.docx]

**Appendix 7: Factors associated with disappearance of neurotoxicity (AFT, N = 23)**

|  | Crude TR (CI 95%) | p | Adj. TR (CI 95%) | p |
| --- | --- | --- | --- | --- |
| Gender  Male  Female | 1  0.67 (0.21-2.15) | 0.51 |  |  |
| Age (in years)  5-11  12-19  20-40  >40 | 3.24 (0.81-12.91)  0.96 (0.21-4.38)  1  0.35 (0.11-1.13) | 0.037 | 1.48 (0.40-5.53)  1.15 (0.32-4.09)  1  0.22 (0.07-0.72) | 0.011 |
| Time since snakebite  [0-2h[  [2h-12h[  [12h-24h[  [24h-48h[  ≥ 48H | 0.25 (0.03-1.76)  0.54 (0.09-3.11)  1  0.18 (0.02-1.79)  3.32 (0.35-31.72) | 0.07 | 0.54 (0.18-1.62)  1  2.75 (0.59-12.90)  1.29 (0.25-6.64)  8.32 (1.72-4.38) | 0.046 |
| Traditional medicine  Yes  No | 1  2.04 (0.62-6.65) | 0.24 |  |  |
| Treatment before arriving at the center  Yes  No | 1  0.70 (0.21-2.34) | 0.56 |  |  |
| Region  North Cameroon  South Cameroon | 1  0.35 (0.12-1.05) | 0.07 |  |  |
| Glasgow score at admission  < 15  15 | 2.28 (0.70-7.40)  1 | 0.17 |  |  |
| Edema before injection  0-1  2  ≥3 | 1  1.45 (0.38-5.63)  1.93 (0.40-9.24) | 0.68 |  |  |
| Hemotoxicity before injection  0  1  ≥2 | 1  0.30 (0.06-1.49)  1.91 (0.49-7.40) | 0.16 |  |  |
|  |  |  |  |  |

TR: Time ratio; CI: confidence interval; BMI: body mass index
