# Supplementary material for: Real life condition evaluation of Inoserp PAN-AFRICA antivenom effectiveness in Cameroon
Source: PLoS Negl Trop Dis. 2023 Nov 8;17(11):e0011707. doi: 10.1371/journal.pntd.0011707 (PMC10659212; doi:10.1371/journal.pntd.0011707)
Supplement: Attachment — Submitted filename: 05_Reviewers ESAA_20230724.docx [file pntd.0011707.s008.docx]

Reviewer's Responses to Questions

Reviewer #1: The objectives are not completely clear and I have some concerns about the study design/sample size and the ethics (see text below)

We thank the reviewer for these helpful remarks. The results presented in this article are those relating to the effectiveness of an antivenom obtained from a study in which the main objective was to assess tolerance. This explains the certain complexity of the presentation especially in terms of design and sample size. We clarified and reorganized the manuscript in line with the reviewer's comments (lines 163-169).

Reviewer #2: Excellent study design given the location in Western Africa for the assessment. Targeting regions with different habitation of venomous snakes of concern is important and was assessed. Population was defined and inclusion and exclusion criteria are reasonable for this investigation. Sample size is also calculated for sufficient power but as author's mentioned, they did not reach their goal, but it did not have an impact on statistical significance.

Some comments and questions to address:

- If patient did not consent but was deemed eligible for study (envenomated) then what was the protocol for clinical care? Please add some details in this situation. Looking at Figure 2, it reveals that 27 individuals were deemed "inappropriate" consent and were excluded. What were the circumstances of why the consenting process was deemed inappropriate. This should be included in the limitations and also in the methods section. Recommend explaining in more detail these two points.

We thank the reviewer for these positive remarks.

We added lines 313-318: “Patients admitted in the participating study sites and who did not consent to participate to the study received care according to the national protocol, i.e. an antivenom available at the Health center level and local treatment. Indications for administration and re-administration were carried out according to national recommendations and were based on exchanges between the health care staff and the bitten patient. No data were collected for patients who did not consent to participate in the study.”

Regarding the 27 "inappropriate" consent, they were related, after on-site monitoring of documents, to patients under 21 years of age (the legal age of majority in Cameroon) for whom the person who signed the consent was found not to be a legal representative according to the Cameroonian laws (e.g. brother, sister, grandparents, aunt/uncle), as well as consents without a legally admissible signature. We haven't added a specific mention because this process is linked to Good Clinical Practice.

Reviewer #3: Line 204 - 207. The additional dose supplied is not described in the text

We thank the reviewer for his remark. We added some details lines 263-254.

--------------------

<b>Results</b></br></br>

-Does the analysis presented match the analysis plan?</br>

-Are the results clearly and completely presented?</br>

-Are the figures (Tables, Images) of sufficient quality for clarity?</br></br>

Reviewer #1: the analysis does not seem to match the objectives. The tables/images/figures are so many that it makes it difficult for the reader to keep track.

We understand the reviewer's point of view. We exposed in this present article results regarding effectiveness and not tolerance. We tried to make the reading as clear as possible. One of the major challenges of this "real-life" study, which took place in very decentralized health centers (where most of the snake-bitten patients were located), was the respect of the recommendations by the health personnel involved in the study. We have been careful to present them, making the reading of the article less fluid but allowing a real representation of the study and therefore of the results interpretation. We clarified and reorganized the manuscript in line with the reviewer's comments.

Reviewer #2: Results are sufficient for analysis. They are presented clearly aside from comment below. Tables and figures are sufficient.

- Line 308 - 311 and table 9, Lines 430 439. I am curious why the individual who was "probably not bitten by a snake" was included in study. Please explain further as it states, "envenomation was excluded". In the clinical history provided it appears to have been reasonable to administer IPA initially but as the case evolved it sounds as if another etiology was at hand. In my opinion, an adjustment may be needed to the mortality calculations to reflect 10 participants. The reason why this is so important is that at least 10 patients did die even after being given IPA via protocol being used. These individuals likely may have needed more vials of antivenom, but this is hard to know at this time. Authors do mention that plasma concentrations of venom were not done and being planned for future study.

We thank the reviewer for his interest and the explanations requested.

Regarding this patient (#15-008-24-NG), based on photos sent afterwards, we observed a single punctate wound on the dorsal side of the foot, without edema or visible hemorrhagic sign. The uniqueness of the inoculation site is not in favor of a snakebite. Patient reported being bitten by a snake while walking at night but did not see the animal responsible for the bite. Patient was treated immediately by traditional medicine with ingestion of plants. He did not present clear clinical signs linked to envenomation. In fact, he did not present symptoms of viperine envenomation (violent pain, edema, hemorrhages, necrosis and normal TCTS) or cobraic (paralysis of the cranial nerves, palpebral ptosis, rictus, muscarinic syndrome, dyspnea).

He presented mainly digestive then respiratory symptoms. Thus, the hypothesis of an intoxication by plants at least as additional factor was retained by the experts.

Given the fact that this patient was included by the doctor-investigator on the inclusion criteria: patient bitten by a snake (declaration not questioned by the investigator) with or without signs of envenomation and faced with the absence of diagnostic certainty and an exposition to the investigated product, we kept this patient in the analysis so as not to risk overestimating the efficacy of the product.

In general, we have chosen to report all serious adverse events occurring among participants included in the study (Lines 464 and following). We have reported one patient who died before product administration but after inclusion (#02-002-06-AB). In our opinion, this exhaustive report provides an accurate presentation of "real-life" local conditions regarding the management of envenomed or suspected envenomed patients. The insufficient number of vials of antivenom administered may be a possible factor for one patient who died (# 05-007-12-AI). For other patients, a long delay between the bite and the start of treatment, inadequate supportive care, especially for hemorrhagic syndromes, such as transfusions of red blood cells or plasma, or support for hemodynamic, cardiac or renal failure, are in our opinion likely to be responsible for the deaths of other patients such as patients 03-018-20-AM, 04-036-45-BA, 04-047-41-YB, 04-054-42-MG, 04-070-25-YD or 04-073-08-FB. Poisoning by traditional medicine products is also a factor that can contribute to death (# 02-084-08-TA).

Reviewer #3: Line 253. 24 unidentified snakes are presented, while in table 2 there are 10

We thank the reviewer and have corrected this error in the table 1.

The text does not show data on blood coagulation values that would allow evidence of its correction in the two hours following the administration of the antivenom.J

As the result of WBCT after antivenom administration was not an indication for a new injection, this data was not systematically collected. We have included the result of the WBCT20 on patient at admission time in the description of population (Lines 389 and following).

--------------------

<b>Conclusions</b></br></br>

-Are the conclusions supported by the data presented?</br>

-Are the limitations of analysis clearly described?</br>

-Do the authors discuss how these data can be helpful to advance our understanding of the topic under study?</br>

-Is public health relevance addressed?</br></br>

Reviewer #1: I wonder if the conclusions can be reorganised and then follow the objectives/results in an easier to read text.

We've tried to clarify to make the text clearer.

Reviewer #2: Conclusions are reasonable and discussed. Each death was also discussed and important. Discussion is thorough and draws on relevant literature. Limitations were discussed and reasonable.

Reviewer #3: No comment

--------------------

<b>Editorial and Data Presentation Modifications?<*/b></br><br/*>

Use this section for editorial suggestions as well as relatively minor modifications of existing data that would enhance clarity. If the only modifications needed are minor and/or editorial, you may wish to recommend “Minor Revision” or “Accept”.

Reviewer #1: (No Response)

Reviewer #2: I recommend accept with minor revision.

Reviewer #3: I suggest that evidence of blood coagulation values be shown. This will make it possible to demonstrate its correction in the two hours following the administration of the antivenom.

--------------------

<b>Summary and General Comments</b></br></br>

Use this section to provide overall comments, discuss strengths/weaknesses of the study, novelty, significance, general execution and scholarship. You may also include additional comments for the author, including concerns about dual publication, research ethics, or publication ethics. If requesting major revision, please articulate the new experiments that are needed.

Reviewer #1: The paper describes the clinical outcomes of a snakebite patient cohort treated with Inoserp antivenom. Data on clinical outcomes are highly needed. But I do have some concerns about this study as it is currently not completely clear which research question the authors are trying to answer and how exactly. I hope the authors can clarify this by reorganizing the data such that the readers can understand the flow better; focusing on their main questions, pulling in the relevant data from the tables/figures in the attachments and remove unneeded tables and analyses irrelevant to the main questions.

In more detail:

-It is not clear which components of the clinical follow-up and therapeutic algorithm in the cohort was part of the standard care in the hospitals involved. Which is relevant to know when interpreting the data.

We exposed the Management algorithm on Appendix 2 and added some sentences to clarify (line 256-258 and 263-264).

-In the introduction it is stated that patients normally have to pay for their antivenom, but in the methods it is explained that antivenom and symptomatic treatment were provided free of charge to the patients in the study. This puts pressure on patients to sign the consent form. Or was antivenom and symptomatic treatment also free for patients who refused to participate? What did the Ethics committees ask about this access to treatment? And how long after the cohort study did thing return to normal (pay for antivenom)? How did that impact health seeking behavior as they were expecting free treatment?

We have taken these ethical aspects into consideration. The study took place in health centers governed by the national health system. The overall objective was to evaluate the efficiency and tolerability of the antivenom, including a clinical follow-up for the participant. In this context, administration and follow up of treatment were under research study responsibility and were free for research participant.

People who refuse to participate received standard care normally provided without this research.

We were aware that this difference could generate undue influence in their participation, however, it did not seem ethical to provide a product under evaluation to a patient that was not included. Moreover, giving free antivenon in the participating health centers could lead to imbalances in the local health system. It should be noted that some persons refuse study participation and preferred to follow regular care (in this case, data were not collected).

According to the international guideline on ethics research involving study participant, an ethical review took place both in the sponsoring institution (Institutional Review Board of the Institut Pasteur) and in the host country (National Ethics Committee of Cameroon). We haven't had any questions or requests for changes regarding the points addressed in the present question.

Moreover, at the end of the study, we made a donation to the Ministry of Public Health of the remaining unused doses. The distribution of this donation was under the responsibility of the Ministry of Public Health, which distributed part to the centers who participated to the study and another part to the other medical centers which take care of envenomed patients.

In addition, in the framework of study, we have set up training courses for the envenomation management as well as a dialogue group allowing local clinicians to benefit from management advices. These advices were provided at the request of the clinicians in charge and concerned patients included or not in the study and could be consider as a collective benefit. These measures put in place have probably improved the management of all patients bitten by snakes who presented to the centers participating in the study.

We added some precision regarding patients who didn’t consent to the study (lines 313-318).

-It is unclear what the main research question was. It switches between effectiveness and tolerability. But effectiveness is not well defined as it’s a cohort. Which is probably why the authors picked adverse effects as the outcome measure used in the sample size calculation. But if that outcome measure adverse effect is used to calculate sample size this is the primary outcome but it’s hardly discussed in the results and discussion and is not in the abstract. Sample size calculation therefore is not in line with the rest of the manuscript.

We clarify that the main objective of this study was tolerability assessment (i.e. calculation of sample size based on this objective) and that we presented in this article the result of effectiveness assessment (lines 166-169). We've deleted any sentences that might have led to confusion about the results presented in the article (section on sample size).

How to define effectiveness of Avs if it’s unknown what would have happened without AV or with an other AV; why not rephrase the manuscript describing outcomes of a patient cohort when AV is used.

It seemed ethically complicated to set up a comparative study against placebo or no specific treatment.

The implementation of a double-blind clinical trial with other antivenoms that do not have a license at the national level appeared complex especially with decentralized health centers. We opted for this type of study design as a first step.

‘Factors associated with early effectiveness were identified using logistic regression models. Didn’t the authors look at factors associated with less severe disease instead of early effectiveness? How to distinguish these two in the current data available? Which variables were tested for effect modification apart from looking at confounders only?

We described severity of envenomation at the time of arrival at Health care center and then evolution of clinical symptoms based on graded assessment scale. We described and evaluated factors associated with improvement of patient health status. Factors associated with less severe disease have already been described in other study. We aimed at describing factors associated with early effectiveness including severity grade, time since snakebite at inclusion due to the fact that patient was referred to health care center after various delay regarding snakebite and thus severity grade. Therefore, factors that may influence lower severity at inclusion were taken into account when we assessed factors associated with early effectiveness. We exposed graded assessment scale (Table 1).

Please use the <https://www.strobe-statement.org/checklists/> to report methodology and results from a cohort.

As explained below, we did not consider this study as a cohort follow-up, but as a prospective clinical study involving a series of patients. The use of STROBE checklist is therefore not justified. We clarified and reorganized the manuscript in order to allow a better understanding of each aspects of the results presented.

The ethics paragraph states there were scientific committees to discuss the results. A representative of the sponsor (Institut Pasteur, Paris) was attending apart from the main investigators from Paris. Who was this representative? As it is also explained that Inoserp financially supports Institut Pasteur, Paris (financial statement: We are grateful to Inosan for providing the antivenoms free of charge and partially funding the study through a partnership with Institut Pasteur, Paris, sponsor of the study). Can the authors explain this partnership? This should probably be reported in the conflict of interest?

The representative of the sponsor was Ms Anais Perilhou.

As described in the paper, Inosan provided the antivenoms free of charge and participated financially in the implementation of the field study.

The contracts between Inosan and the Pasteur Institute guaranteed the total independence of the study team in the execution of the study and the analysis of the data.

The terms of this contract were presented to the various ethics committees that approved the project. No member of the study team received direct funding from Inosan. This situation does not constitute a conflict of interest and has been clearly explained to the ethics committees and in the article presented.

I am confused by the different numbers. Eg In the results there are 336 cytotoxic symptoms with at least one antivenom injection, in the abstract in 346 and in the table 5 cytotoxic only 108 + cytotoxic and bleeding 234 is together 342. Do the authors think the model they developed to look at effectiveness works for the 108 patients with cytotoxic problems?

We understand the reviewer’s concerns and we thank him for his comments. The main difficulties were the presentation of results according to the populations concerned. We have described the overall population of patients included in the study (n=447), and among them the envenomed patients (n=369, figure 2 and 3), patients who received AV (n=362, Table 2) and envenomed patients who received at least one antivenom administration (n= 356, Table 2 and Figure 3). All were described Table 2.

We performed the effectiveness analysis on the population of patients with SBE who had received at least one dose of antivenom (n=356 minus one participant who left hospital against medical advice before the post-injection clinical evaluation but who has been evaluated at long-term visit ) as exposed in the Statistical analysis section, lines 270-272: “*Effectiveness was investigated in all patients with envenomation symptoms SBE who received at least one antivenom injection and who had at least one clinical evaluation following the injection.*”

We have corrected errors and clarified the text of the summary and results to better address your concerns.

We have revised the abstract by focusing the description on the population of patients with SBE who have received at least one dose of antivenom.

Figure 3: We corrected the number of patients without antivenom (corrected number: 13; 85 corresponding to overall patients without antivenom administration).

Table 4: We added the words “among patients who received antivenom” in the title and we have added descriptive lines to clarify the number of people per clinical syndrome, which can be unique or a combination of syndromes.

We added a sub-title section in the results “*Patients presenting SBE and at least one antivenom administration*”.

Regarding cytotoxic signs, we clarify that this criterion was not considered for early effectiveness evaluation (line 275-276). We also described the evolution of cytotoxic signs on lines 372-386 and figure 4.

I have challenges reading the discussion; can authors remove info on patient outcomes/case histories to the results and move p=values (or preferably effect measures) to the results? Referring to tables in the discussions makes it also more difficult for the reader to follow the main messages.

We moved case histories to the results and removed p values and referring to tables in the discussion as asked by the reviewer.

Title: Why is there a ‘field evaluation’ mentioned in the title whereas it is all about hospital treatment?

We changed "field evaluation" by "real life condition". We have used this expression to emphasize the absence of dedicated capacity building in terms of medical logistics and medical staff within the framework of the study. In addition, the study took place in highly decentralized care centers, where the majority of patients were treated. Many similar studies take place in specialized centers in cities with large populations. We thought it would be useful to point this out.

Abstract:

Line 26 However: what is the contrast? And mentioned is effectiveness urgently needed and then the next line ‘ we assessed tolerance and effectiveness’. Not clear; is your main goal tolerance or effectiveness?

We corrected and clarified following reviewer’s recommendations.

Study design is not included (cohort study). Explain standard practice versus procedures followed in cohort.

We did not consider this study as a cohort follow-up, but as a prospective clinical study consisting in monitoring the treatment administered and its impact during the few days of hospitalization and a few weeks after discharge. This is described in the materials and methods section.

Regarding standard practices versus procedures of the study, we have added a few clarifications in lines 215-219.

Introduction:

Line 100-101 ‘the main problem encountered in SSA is the poor accessibility to antivenoms’. That will depend per country. In many countries/regions the availability is worse than the accessibility.

We thank the reviewer for this relevant comment, with which we fully agree. However, there is a close link between availability and accessibility of antivenoms in sub-Saharan Africa, as explained in references 6, 10, 11 and 12 (that we added in the main text). It's a "vicious circle" that starts with the high price of antivenoms (mainly at the end of 90s), which limited antivenom accessibility. This leads to a drop in purchases, and therefore in manufacturing, which in turn results in poor availability in some parts of sub-Saharan Africa. We have, however, modified the sentence by adding the issue of availability to take account of the reviewer's comment (Lines 112-113.

Line 109 ASS (French)

We corrected.

Some more data from ref 14 and 15 on Inoserp is needed which can also help us to understand which data is missing from these cohorts which will be obtained in the current study.

We added more details line 128-158.

Authors state that ‘patients refuse to buy more than one or two vials at the time’. That cannot be generalized for entire SSA.

We deleted the sentence

Methods: clinical study -> cohort

We answered this remark previously.

Why are patients younger than 5 years excluded?

Due to the lack of data for this age group, the sponsor did not wish to include them, despite the absence of contraindication provided by the manufacturer.

Line 186 *E ocellatus* -> *romani*

This section corresponding to the description in the manufacturer's leaflet, we have kept the denomination as it was written.

Assessment of clinical outcomes: what if patients initially improve but then deteriorate?

In the event of secondary deterioration, the same dose as initially was renewed in the patient. This point has been clarified in the text (line 261 and following).

Statistical analysis: all 356 -> number of patients included in the methods should move to the results instead of being in the methods section.

We corrected.

Please discuss the validity of the definition of ‘early effectiveness/medium-term effectiveness’ model used.

We have added explanations on this point (lines 274 and following).

Line 230: 'some ad hoc analyses' is too vague. And there are many ad hoc analyses in the appendices; can you reduce the number of analyses based on the research questions to avoid unexpected, biologically difficult to explain p-values?

We thank the reviewer for his remark. We performed various analysis which are not presented in the present paper. As the consequence, we deleted this sentence that didn’t make sense here.

Ethics: please add the numbers of the ethics evaluations.

We added details requested (line 309-312).

Results line 154; please provide RR + intervals instead of p-values.

We corrected.

Lines 264-266: figure briefly describes clinical presentation of the patients, including death? Now reads as if patients came in dead.

We removed patient evolution from this flowchart

The first line in the results/effectiveness assessment is on the storage conditions of IPA instead of a topic related to effectiveness?

We removed this sentence.

Line 287: ‘Improvement was noted within 2 hours after the first antivenom administration in 189 patients. This means a positive 20WBCT switched to negative? No INR data? How many missing 20WBCT do you have (as reported in the discussion as a limitation)? How many had a positive 20WBCT, then negative and then back to positive?

Improvement was more clearly defined (line 270-277): decrease of at least 1 grade (clinical evaluation) between two time evaluation.

20WBCT was used at the time of initial evaluation. This test is not a criteria for renewal of antivenom administration. We added some data regarding 20WBCT results.

Line 289: ‘ complete stop of bleeding was achieved within 2 hours’. How was this defined? As it can be quite difficult to know for sure a bleeding stopped within 2 hours? Minor or major bleedings?

It corresponds to Grade 0 of bleeding gradation performed by clinical investigators during follow-up as for initial evaluation. We clarified line 405.

Line 299: Multivariate analysis time between snakebite and antivenom injection was significantly associated with early improvement… and then a p-value follows instead of an effect size (OR + interval). Please report results in line with multivariate analysis instead. Another question on this analysis is; The cytotoxic patients presented later and it takes more time to recover. So how do you know you aren’t finding the cytotoxic patient effect? Any effect modification based on the clinical syndrome patients present with in the model?

This point has been re-assessed and described in the text (Lines 373 and following).

Bleeding grades in the multivariate analysis (303) were associated with lower odds; please present as normally done in a multivariate analysis assuming the odds is lower but not below 1..?

We clarified and precised that numbers 1 to 3 correspond to symptoms gradation.

Line 311: why were patients included who probably did not have a snakebite?

Only one patient corresponds to this description. We tried to describe his clinical history. However, this patient was deemed to have been bitten by a snake by the clinical investigator and received antivenom. We have chosen to include him in the analysis to avoid the risk of underestimating any serious adverse events that may have occurred in the framework of this study. This point has been detailed in the text (Lines 470 and following).

Line 313: who decided the scars were unsightly? And who decided the amputations were minimal and the functional limitations? Patient reported?

Clinical investigators reported clinical evaluation after interview and clinical exams of patients. These assessments were performed on an independent basis, as for a medical assessments of disability with a medico-legal aspect.

Discussions: Line 322 – 352 are not related to the study findings and read as a general background.

We agree with the reviewer's very fair comment. We reorganized the manuscript as requested.

Line 397: female gender was associated,…. We did not have a clear explanation for this finding. Which leads to the earlier comment/question asking the authors to answer the primary research question and some secondary questions but reduce the post hoc analyses?

We explained that we did not see clear explanation to this association. We clarified the manuscript following reviewers’ recommendations.

How do authors know the study also confirms that IPA no more than other antivenoms is unable to stop or slow the progression of edema? There was no comparison to other antivenoms and the follow-up method seemed not adapted to assess impact on cytotoxic lesions as these focused on bleeding and neurotoxicity.

We did not conclude on the effectiveness of the antivenom in comparison with others, as this would be inconsistent with the design of the study presented.

We describe points evaluated and explain the difficulty of assessing the evolution of edema. We also explain that the evolution of clinical signs depends not only on the antivenom, but also on the associated supportive care, which is also valid for edema.

Author contributions:

Can authors explain the author contributions a bit more? The study took place in Cameroon but the conceptualization and methods were developed by two team members in Paris only. Was there no codevelopment with the team in Cameroon? And the only software used is STATA and REDCaP but one coauthor has software as the only role?

We thank the reviewer for his remarks. We mistakenly omitted AN's contribution to Conceptualization, Methodology. This has been corrected. Regarding MS, this refers to a contribution to Data Management.

Baseline factors associated with neurotoxicity N-23 but there are 21 variables in the model and many of them require dummy variables which further hampers power. Which power did the authors expect with this combination? With such limited power, why do you add the different first line home treatments whereas that question does not fit one of your main research questions (outcomes after AV and tolerability of AV?) Same applies to the model with haematotoxic ‘troubles’?

Regarding neurotoxicity, this is the reason why we only conducted a univariate analysis. We did not have the statistical power to evaluate the effects of several factors at the same time.

None of the other factors investigated were significantly associated with improvement of neurotoxicity. Treatments such as traditional medicine received prior to hospital arrival have been taken into account, as they may have neurotoxic or haematotoxic effects and influence the patient's clinical evolution

Reviewer #2: Authors have conducted a thorough investigation of IPA in SSA. These findings are important to the region and the results are promising. Some minor comments are found which I believe can be addressed for clarity.

Reviewer #3: The article is interesting, however it is necessary to clarify how the coagulation correction was evidenced at two hours taking into account the absence of initial values and two hours later since it does not show laboratory data

We thank the reviewer for his comment. We added the 20WBCT data we had available (Table 4 and lines 388-390). This study was carried out in real-life conditions and in decentralized medical centers, with no laboratory facilities able to analyze coagulation disorders. We report here the results relating to the standard management of snakebites in this area.
